# Supplementary material for: Soil properties and plant functional traits have different importance in shaping rhizosphere soil bacterial and fungal communities in a meadow steppe
Source: mSystems. 2025 Jul 8;10(8):e00570-25. doi: 10.1128/msystems.00570-25 (PMC12363235; doi:10.1128/msystems.00570-25)
Supplement: Supplemental material — Table S1–S5; Fig. S1–S13. [file msystems.00570-25-s0001.docx]

**Supplemental figures and tables**

**Table S1** Soil properties and plant functional traits used in the analysis.

| **Tissue** | **Variables** | **Abbreviation** | **Unit** |
| --- | --- | --- | --- |
| **Soil** | Soil water content | SWC | % |
|  | hydrogen ion concentration | pH | - |
|  | Soil organic carbon | SOC | g/kg |
|  | Total nitrogen | TN | g/kg |
|  | Available phosphorus | AP | mg/kg |
|  | Total phosphorus | TP | g/kg |
|  | Ammonium nitrogen | NH_4_^+^ | mg/kg |
|  | Nitrate nitrogen | NO_3_^-^ | mg/kg |
| **Leaf** | Leaf thickness | LT | mm |
|  | Specific leaf area | SLA | cm^2^ mg^-1^ |
|  | Leaf dry matter content | LDMC | % |
|  | Leaf mass per area | LMA | g m^-2^ |
|  | Leaf carbon content | LC | % |
|  | Leaf nitrogen content | LN | % |
|  | Leaf phosphorous content | LP | g/kg |
|  | Leaf carbon nitrogen ratio | LCN | - |
| **Root** | Specific root area | SRA | cm^2^ mg^-1^ |
|  | Specific root length | SRL | cm mg^-1^ |
|  | Root tissue density | RTD | kg m^-3^ |
|  | Average root diameter | RD | mm |
|  | Branching intensity | BI | forks cm^-1^ |
|  | Specific root tip abundance | SRTA | tips mg^-1^ |
|  | Root carbon content | RC | % |
|  | Root nitrogen content | RN | % |
|  | Root phosphorous content | RP | g/kg |
|  | Root carbon nitrogen ratio | RCN | - |
|  | Root depth | D | cm |
| **Plant community** | Average height | Hei | cm |
|  | Relative cover | Cov | % |
|  | Species identity | P | - |

**Table S2** Permutational multivariate analysis of variance (PERMANOVA) to test the significance of differences in bacterial and fungal community composition between sites. *R^2^* and *P*: PERMANOVA test statistic.

|  | **pairs** | ***R^2^*** | **p.adjisted** |
| --- | --- | --- | --- |
| **Bacteria** | **All** | 0.066 | **<0.001** |
|  | **Site A vs Site B** | 0.009 | 0.472 |
|  | **Site A vs Site C** | 0.079 | **0.002** |
|  | **Site B vs Site C** | 0.070 | **0.002** |
| **Fungi** | **All** | 0.034 | **<0.001** |
|  | **Site A vs Site B** | 0.024 | **0.002** |
|  | **Site A vs Site C** | 0.039 | **0.002** |
|  | **Site B vs Site C** | 0.015 | **0.021** |

**Table S3** One-way ANOVA between sites for the relative abundance of different phyla taxa. Statistical differences indicated by post hoc Tukey HSD test are represented in different lowercase letters.

|  |  |  |  |  | **Between sites** | | |
| --- | --- | --- | --- | --- | --- | --- | --- |
|  | **Taxa** | **Site A** | **Site B** | **Site C** | **df** | ***F* value** | ***P*** |
| **Bacteria** | **Proteobacteria** | 27.07±4.52 ^b^ | 27.45±5.67 ^b^ | 32.67±5.61 ^a^ | 2 | 15.24 | **<0.001** |
|  | **Acidobacteria** | 16.70±3.07 ^a^ | 16.78±2.96 ^a^ | 16.24±2.93 ^a^ | 2 | 0.419 | 0.658 |
|  | **Bacteroidetes** | 14.78±4.37 ^a^ | 13.36±3.23 ^a^ | 14.71±4.77 ^a^ | 2 | 1.864 | 0.159 |
|  | **Actinobacteria** | 14.40±3.72 ^a^ | 15.12±4.08 ^a^ | 13.82±4.47 ^a^ | 2 | 1.162 | 0.316 |
|  | **Planctomycetes** | 7.52±1.20 ^a^ | 7.54±0.93 ^a^ | 6.37±0.99 ^b^ | 2 | 17.54 | **<0.001** |
|  | **Chloroflexi** | 5.96±1.14 ^a^ | 5.76±1.10 ^a^ | 5.06±0.90 ^b^ | 2 | 8.573 | **<0.001** |
|  | **Verrucomicrobia** | 4.77±2.56 ^a^ | 5.11±2.95 ^a^ | 4.42±3.50 ^a^ | 2 | 0.598 | 0.551 |
|  | **Thaumarchaeota** | 3.23±2.00 ^a^ | 3.05±2.30 ^a^ | 1.75±1.67 ^b^ | 2 | 0.815 | **0.002** |
|  | **Gemmatimonadetes** | 1.45±0.37 ^a^ | 1.56±0.29 ^a^ | 1.50±0.34 ^a^ | 2 | 1.279 | 0.281 |
|  | **Armatimonadetes** | 0.52±0.14 ^a^ | 0.50±0.17 ^a^ | 0.38±0.14 ^b^ | 2 | 10.45 | **<0.001** |
| **Fungi** | **Ascomycota** | 61.79±16.77 ^a^ | 55.23±18.84 ^a^ | 62.53±18.20 ^a^ | 2 | 2.526 | 0.084 |
|  | **Basidiomycota** | 26.86±17.18 ^a^ | 29.74±20.44 ^a^ | 12.03±13.15 ^b^ | 2 | 13.16 | **<0.001** |
|  | **unidentified** | 0.54±0.57 ^a^ | 0.64±0.77 ^a^ | 0.69±1.07 ^a^ | 2 | 0.416 | 0.66 |
|  | **Glomeromycota** | 2.32±2.24 ^a^ | 1.69±2.23 ^a^ | 0.18±0.16 ^b^ | 2 | 14.91 | **<0.001** |
|  | **Mortierellomycota** | 1.86±6.13 ^b^ | 5.24±11.35 ^b^ | 13.38±15.30 ^a^ | 2 | 12.35 | **<0.001** |

**Table S4** The standardized variable loadings for leaf or root functional traits PC1 and PC2. The detailed description of each plant functional traits is presented in Table S1.

| Leaf functional traits | | | Root functional traits | | |
| --- | --- | --- | --- | --- | --- |
| Variables | PC1_Leaf_ | PC2 _Leaf_ | Variables | PC1_Root_ | PC2 _Root_ |
| LC | 0.012 | 0.906 | RC | 0.157 | 0.484 |
| LCN | -0.831 | -0.026 | RCN | 0.435 | 0.848 |
| LMA | 0.851 | 0.209 | RTD | -0.594 | 0.518 |
| LDMC | 0.741 | 0.266 | RP | -0.366 | -0.666 |
| SLA | 0.886 | -0.162 | RN | 0.340 | -0.756 |
| LP | 0.865 | 0.128 | RD | -0.680 | 0.303 |
| LN | 0.808 | 0.464 | BI | 0.615 | 0.034 |
|  |  |  | SRA | 0.898 | -0.308 |
|  |  |  | SRL | 0.944 | -0.228 |
|  |  |  | SRTA | 0.905 | 0.043 |

**Table S5** Forward selection of twenty-nine variables (eight leaf functional traits, 11 root functional traits, two community traits, eight soil properties) on bacterial and fungal OTU sequence count matrix. The detailed description of each soil properties and plant functional traits is presented in Table S1.

| **Bacteria** | | | | | | **Fungi** | | | | | |
| --- | --- | --- | --- | --- | --- | --- | --- | --- | --- | --- | --- |
| **Variables** | ***R^2^*** | ***R^2^Cum*** | ***Adj.R^2^Cum*** | ***F*** | ***P* value** | **Variables** | ***R^2^*** | ***R^2^Cum*** | ***Adj.R^2^Cum*** | ***F*** | ***P* value** |
| **SWC** | 0.055 | 0.055 | 0.048 | 8.151 | 0.001 | **SWC** | 0.044 | 0.044 | 0.038 | 6.667 | 0.001 |
| **D** | 0.034 | 0.089 | 0.076 | 5.198 | 0.001 | **D** | 0.021 | 0.065 | 0.052 | 3.175 | 0.001 |
| **RN** | 0.016 | 0.105 | 0.086 | 2.485 | 0.001 | **LP** | 0.013 | 0.078 | 0.059 | 2.034 | 0.001 |
| **TN** | 0.015 | 0.120 | 0.094 | 2.311 | 0.002 | **AP** | 0.012 | 0.091 | 0.065 | 1.927 | 0.001 |
| **LT** | 0.014 | 0.134 | 0.102 | 2.173 | 0.004 | **NO_3_^-^-N** | 0.012 | 0.103 | 0.070 | 1.856 | 0.001 |
| **LC** | 0.010 | 0.144 | 0.106 | 1.577 | 0.028 | **TP** | 0.011 | 0.114 | 0.075 | 1.721 | 0.001 |
| **SLA** | 0.010 | 0.154 | 0.109 | 1.525 | 0.042 | **SOC** | 0.013 | 0.126 | 0.082 | 2.046 | 0.001 |
| **LP** | 0.011 | 0.164 | 0.114 | 1.727 | 0.012 | **TN** | 0.012 | 0.138 | 0.088 | 1.883 | 0.001 |
| **SOC** | 0.009 | 0.173 | 0.117 | 1.452 | 0.049 | **RN** | 0.011 | 0.149 | 0.093 | 1.714 | 0.001 |
|  |  |  |  |  |  | **LT** | 0.009 | 0.158 | 0.096 | 1.431 | 0.010 |
|  |  |  |  |  |  | **SLA** | 0.009 | 0.167 | 0.098 | 1.380 | 0.016 |
|  |  |  |  |  |  | **LDMC** | 0.010 | 0.176 | 0.102 | 1.537 | 0.002 |
|  |  |  |  |  |  | **NH_4_^+^-N** | 0.009 | 0.185 | 0.105 | 1.423 | 0.008 |
|  |  |  |  |  |  | **pH** | 0.016 | 0.201 | 0.116 | 2.658 | 0.001 |


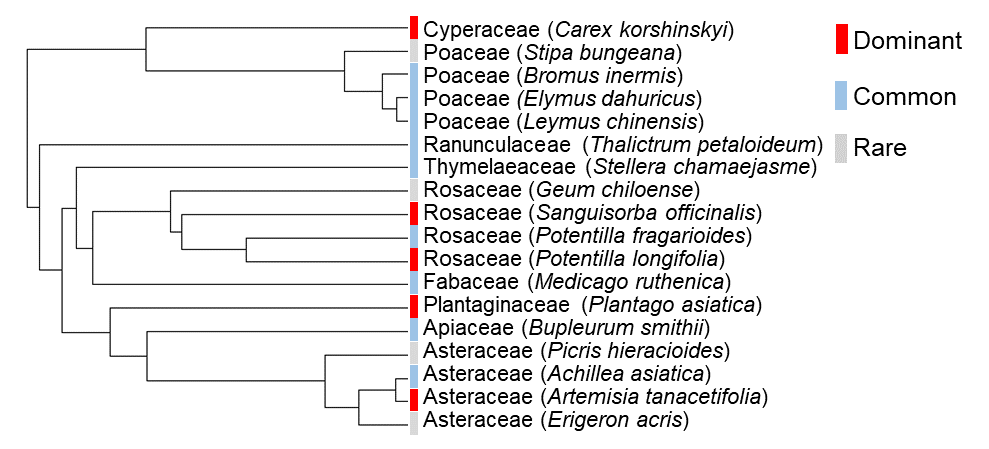


**Figure S1** Phylogenetic tree of 18 species involved in this study.


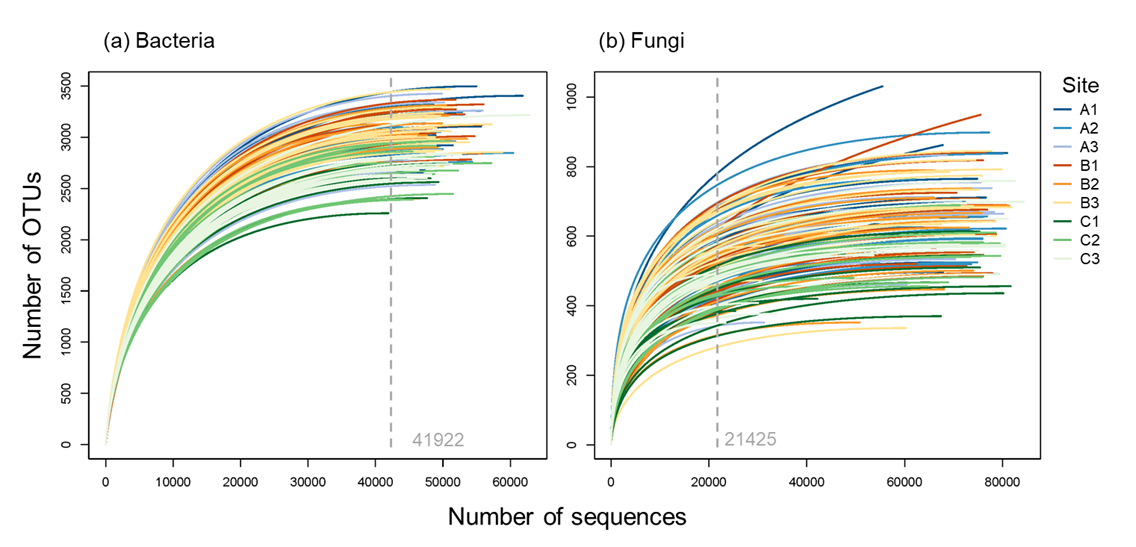


**Figure S2** Rarefaction curves of bacteria (a) and fungi (b) in rhizosphere soil. The vertical dashed line represents the lowest sequence number of the original sample, based on which all samples were diluted to control for the sampling effort.

**
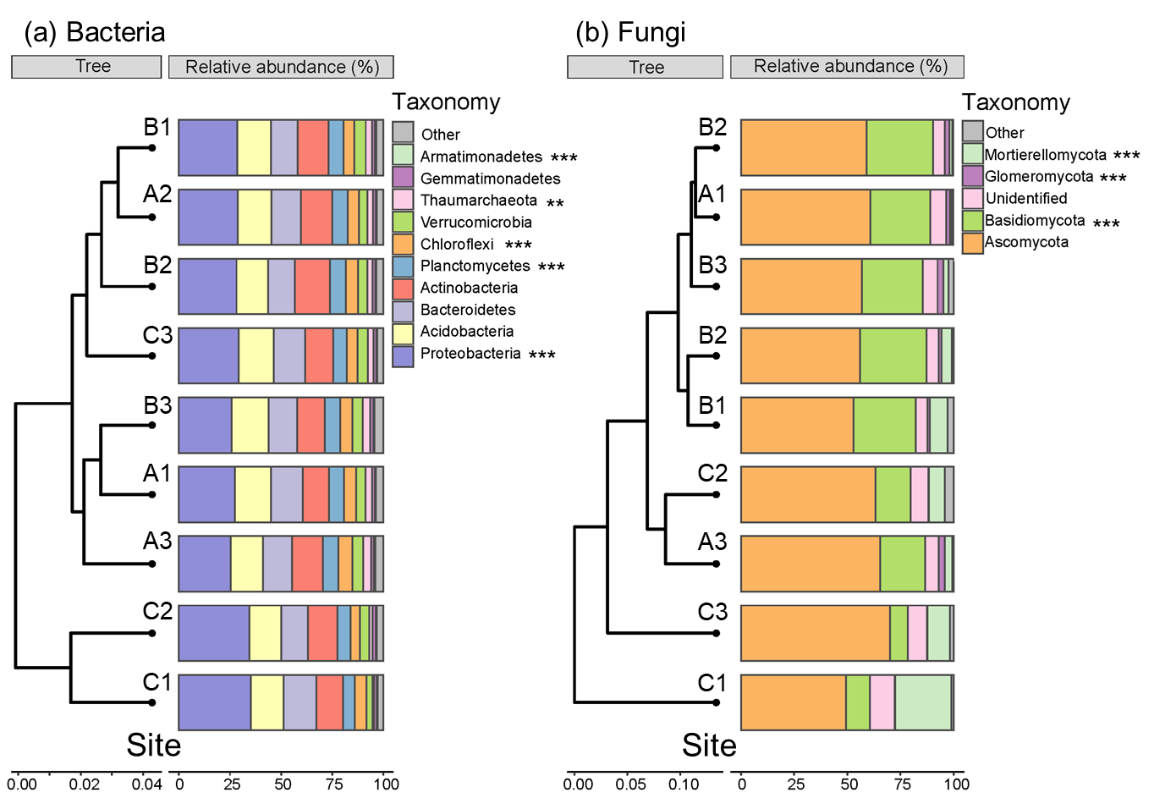
**

**Figure S3.** Relative abundance of different phyla in bacteria (a) and fungi (b). The dendrograms indicate the similarities in OTU composition among different sites according to the average-linkage clustering results of the Bray-Curtis distance matrix. Asterisks indicate that the relative abundance of taxa differ statistically between sites. * : *P* < 0.05; ** : *P* < 0.01; *** : *P* < 0.001.


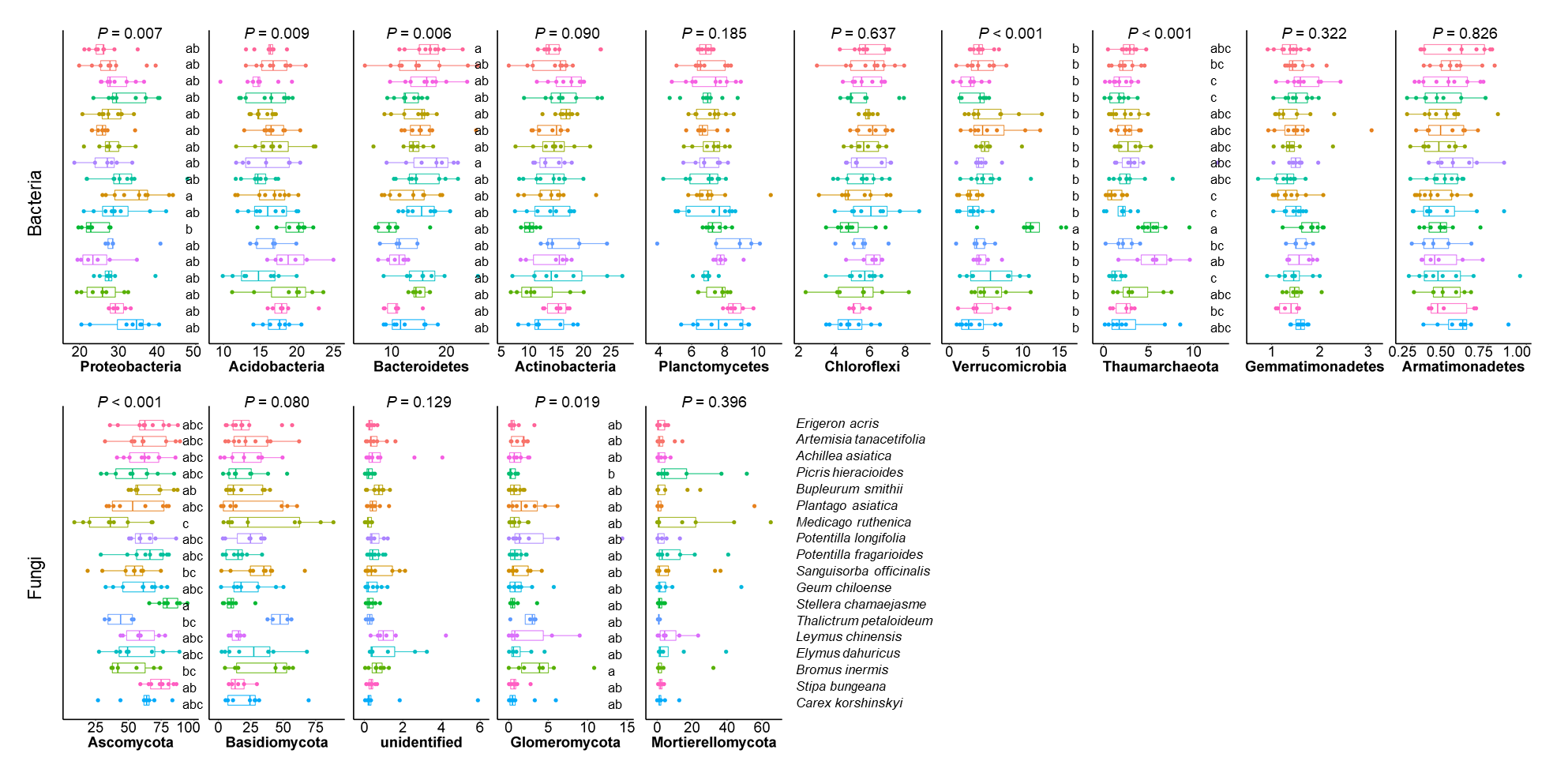


**Figure S4** Relative abundance of different phyla taxa (%) in bacteria and fungi. Statistical differences indicated by post hoc Tukey HSD test are represented in different lowercase letters.


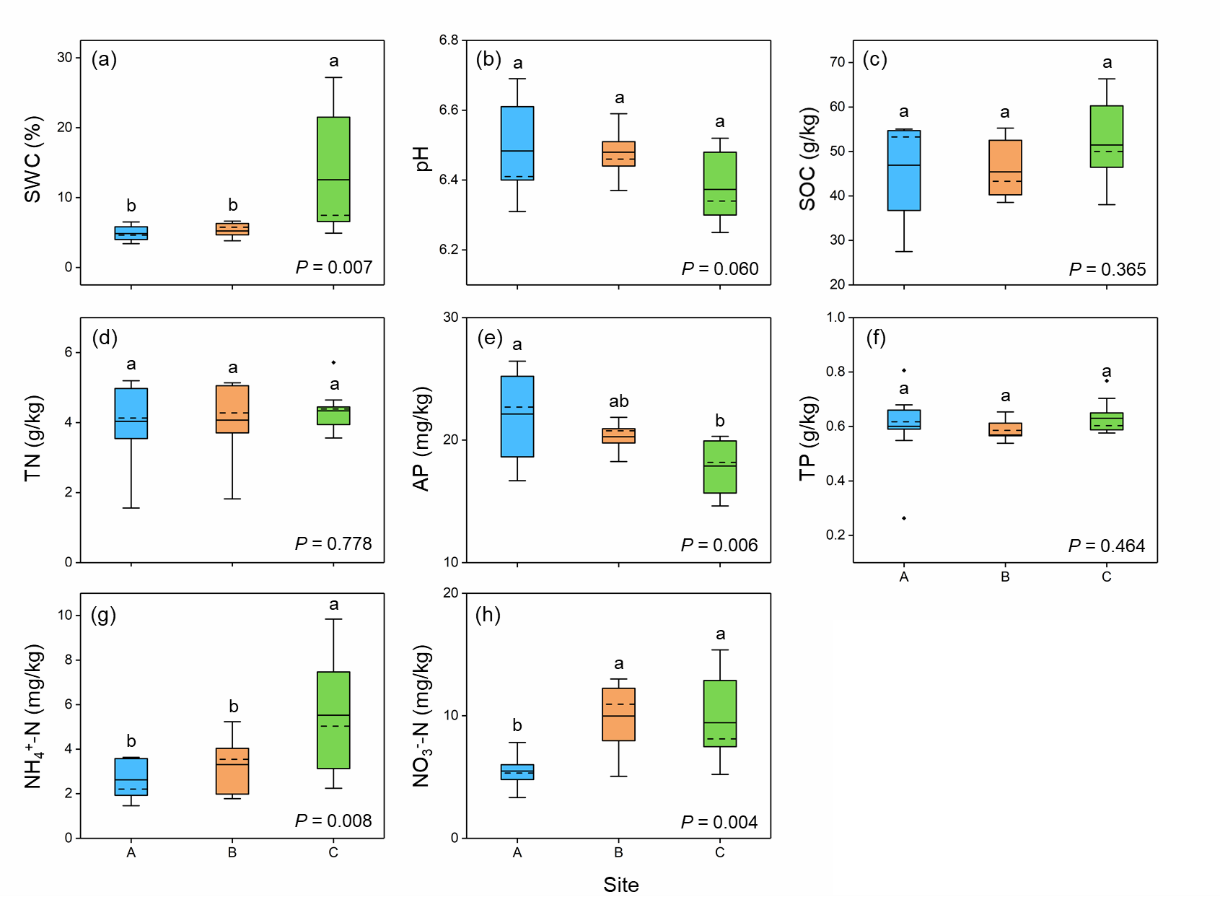


**Figure S5** Variations of soil water content (a, SWC), hydrogen ion concentration (b, pH), organic carbon concentration (c, SOC), total nitrogen concentration (d, TN), available phosphorus concentration (e, AP), total phosphorus concentration (f, TP), ammonium nitrogen concentration (g, NH_4_^+^-N) and nitrate nitrogen concentration (h, NO_3_^-^-N) between sites (*n* = 9). The solid and dashed lines in box plots represent the mean and median values, respectively. Lowercase letters represent the differences between sites (*P* < 0.05; post hoc Tukey HSD test).


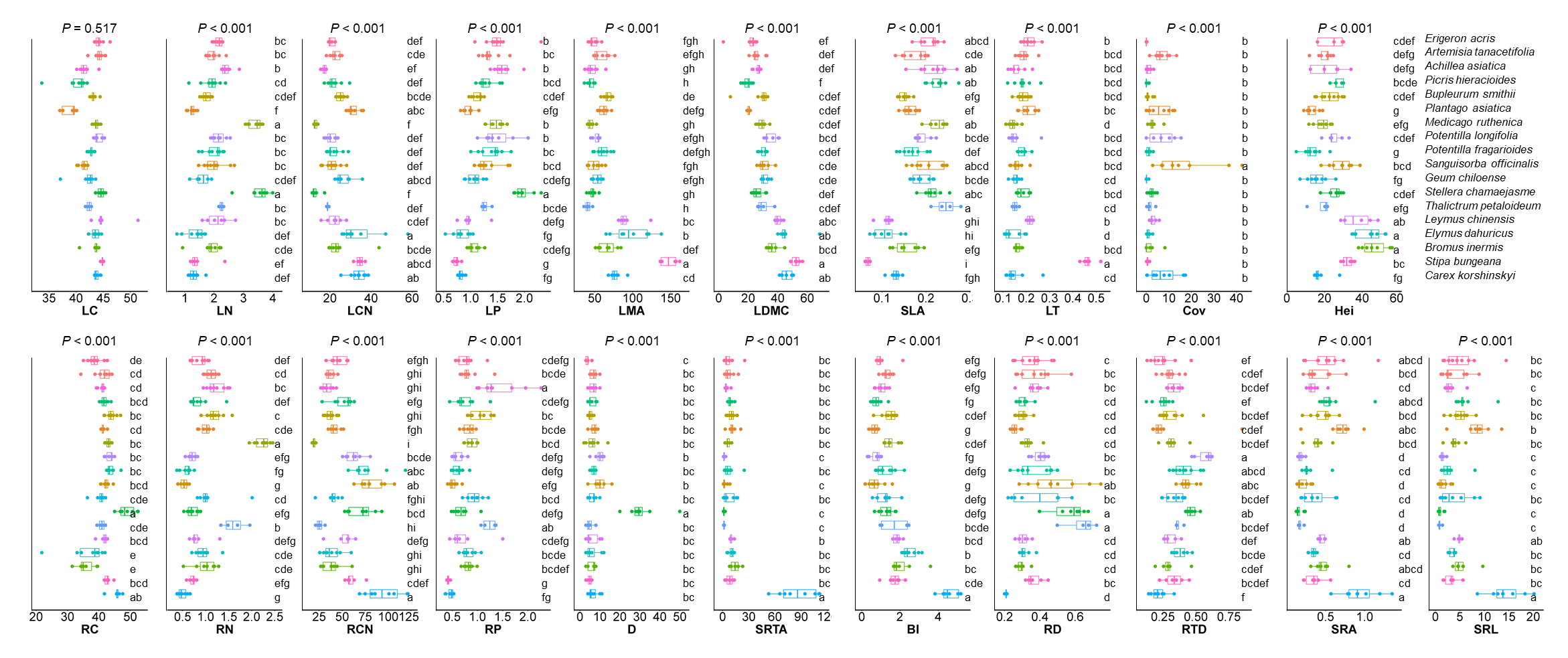


**Figure S6** Plant functional traits of each species. Statistical differences indicated by post hoc Tukey HSD test are represented in different lowercase letters. The detailed description of each plant functional traits is presented in Table S1.


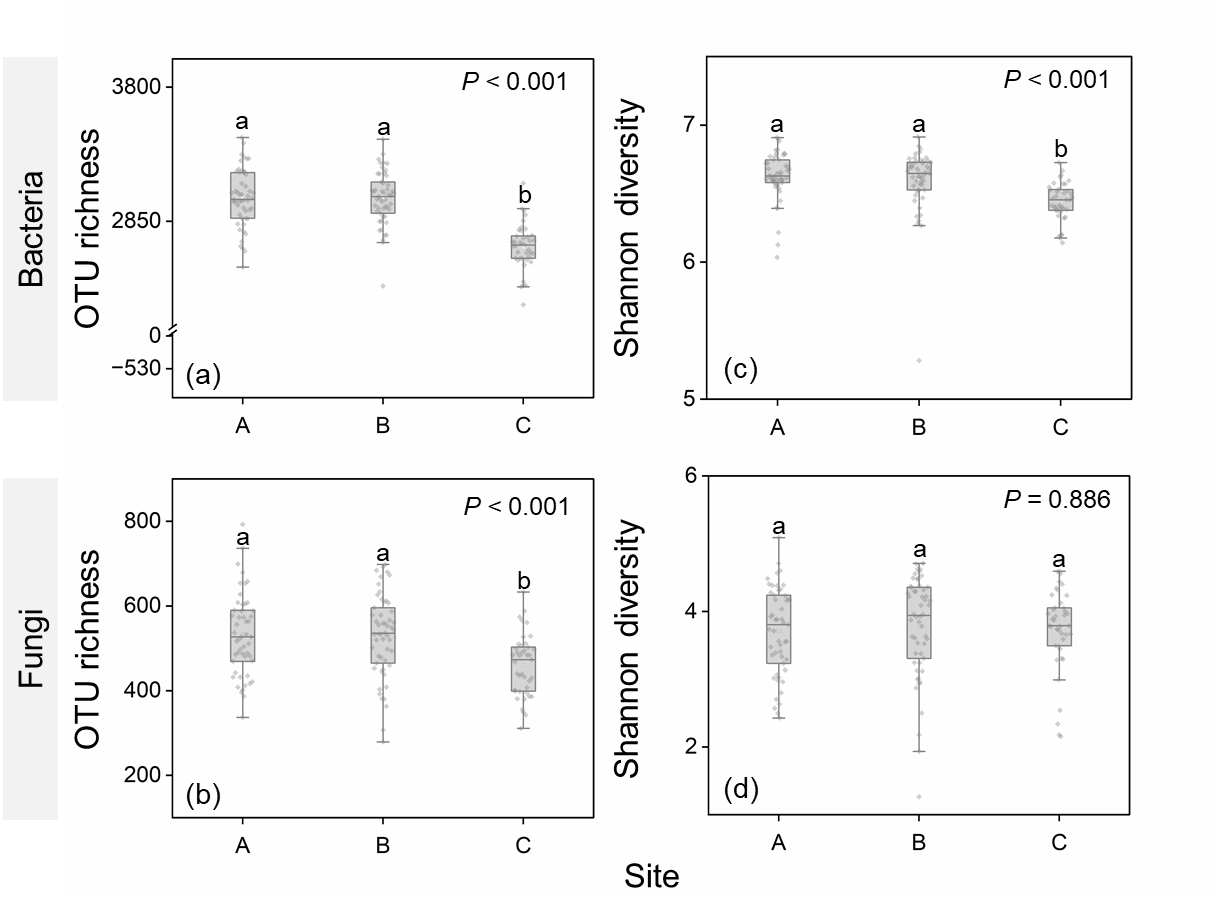
 **Figure S7** OTU richness and Shannon diversity of rhizosphere soil bacteria (a, c) and fungi (b, d) in different sites. Statistical differences indicated by post hoc Tukey HSD test are represented in different lowercase letters.


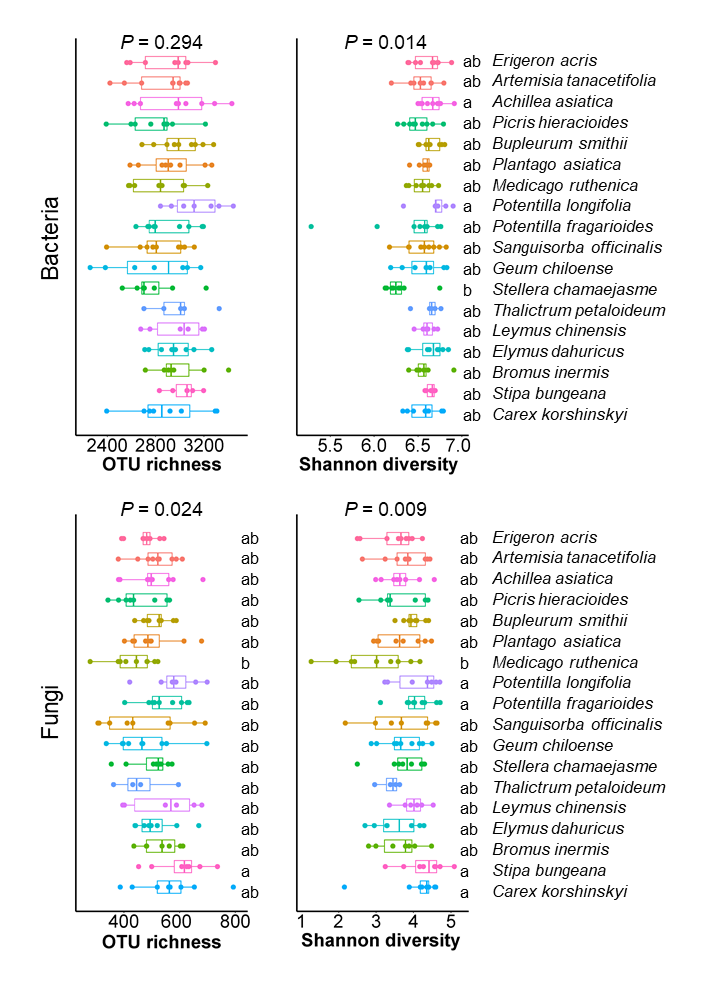


**Figure S8** OTU richness and Shannon diversity of rhizosphere soil bacteria and fungi in different species. Statistical differences indicated by post hoc Tukey HSD test are represented in different lowercase letters.


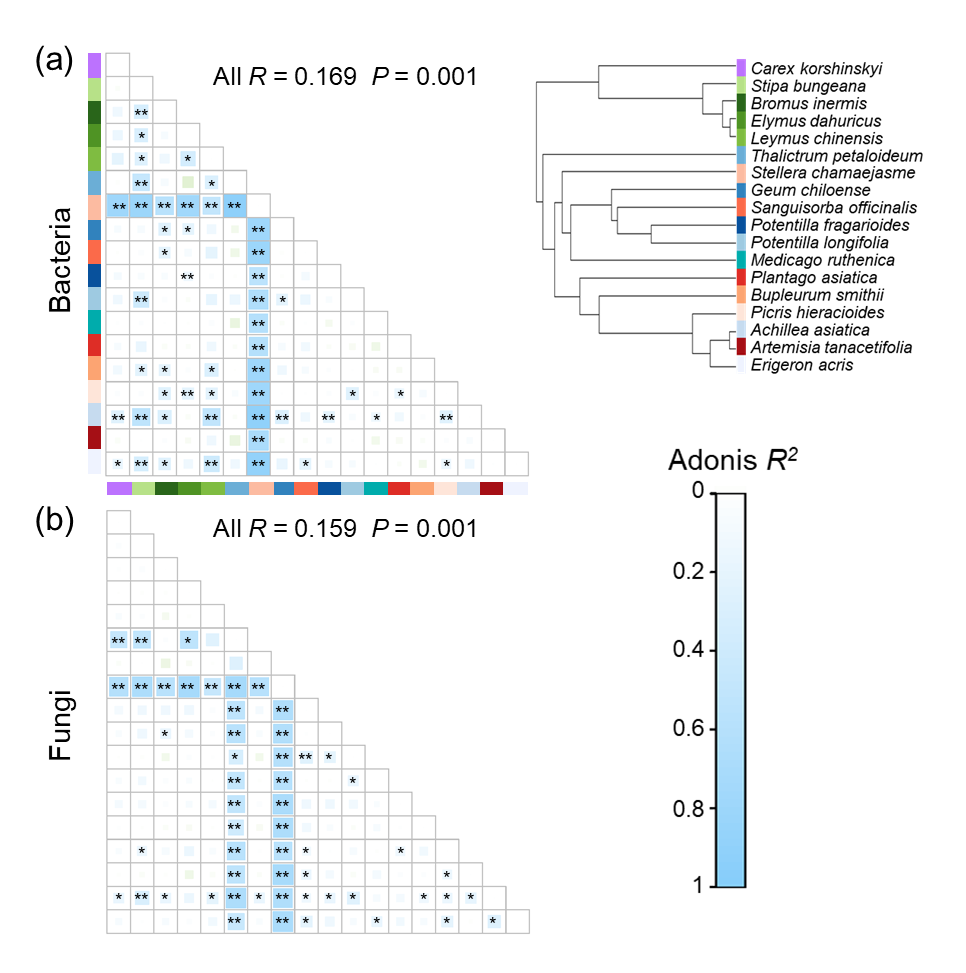


**Figure S9** Permutational multivariate analysis of variance (PERMANOVA) to test the significance of differences in bacterial (a) and fungal (b) community composition between species. The *R^2^* values of the color gradient represents the degree of explanation for differences in microbial community composition between paired species, and the asterisk represents the statistical *P* values based on 999 permutations. * : *P* < 0.05; ** : *P* < 0.01.


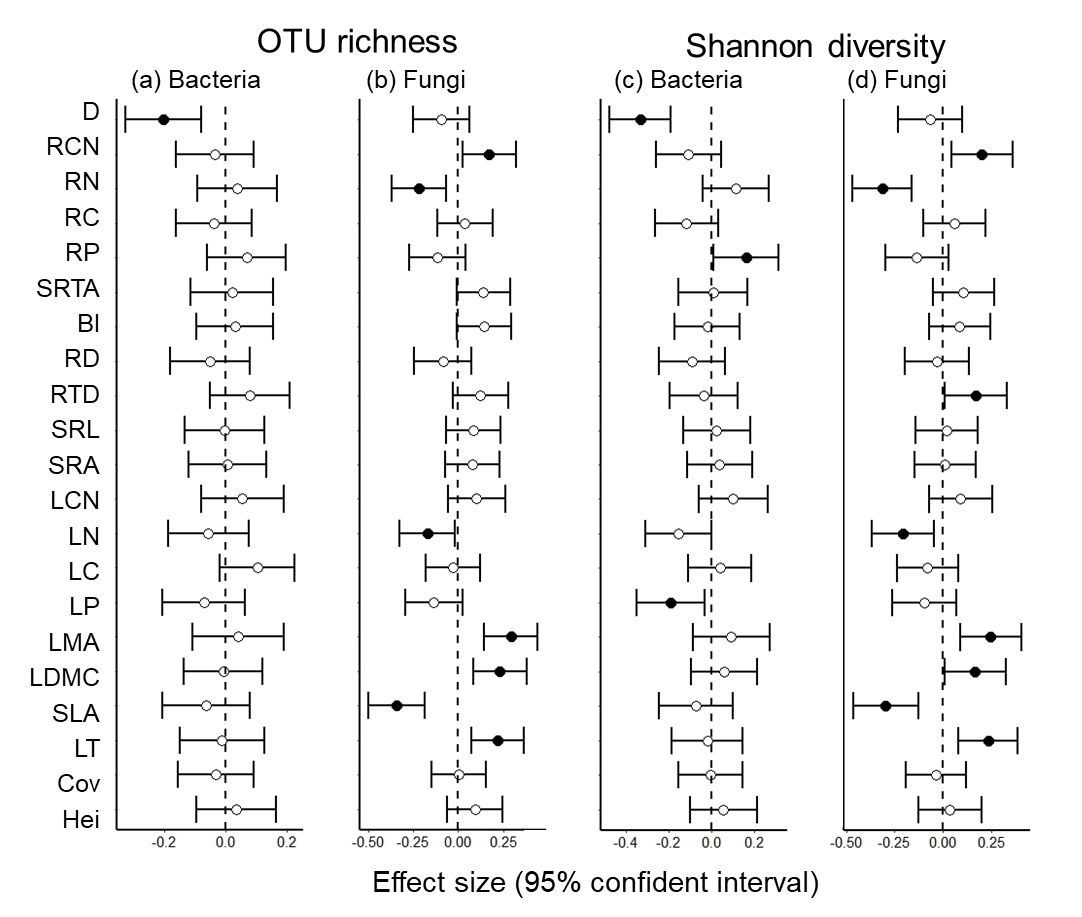


**Figure S10** The linear mixed-effects models of the effects of plant functional traits on rhizosphere soil bacterial and fungal diversity. Effect sizes are standardized coefficients estimated separately for each predictor variable in simple linear models. Solid and open circles indicate significant and insignificant effects, respectively. The detailed description of each soil properties and plant functional traits is presented in Table S1.


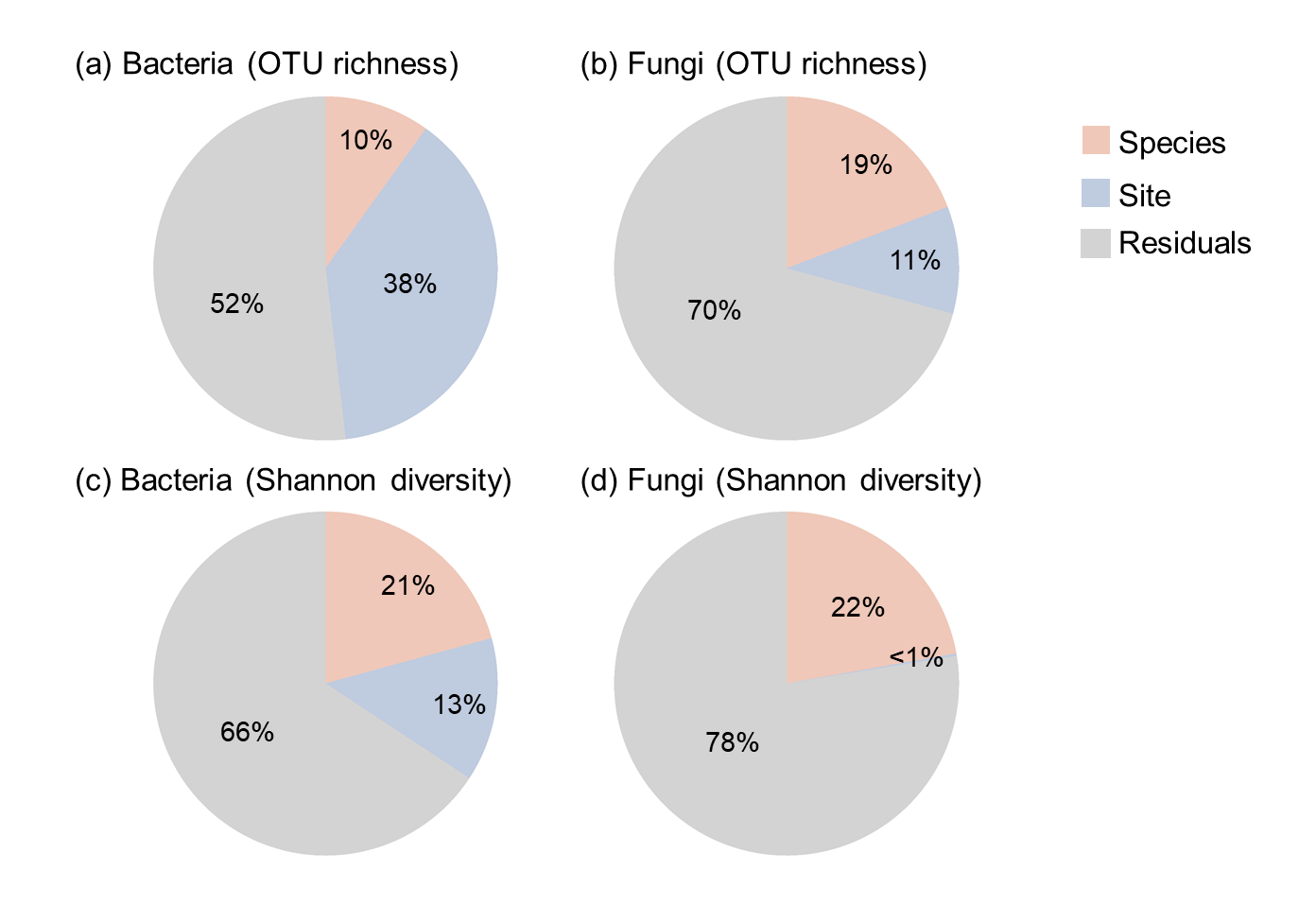


**Figure S11** Proportion of variance in bacterial (a, c) and fungal (b, d) diversity explained by plant species, site, and residuals.


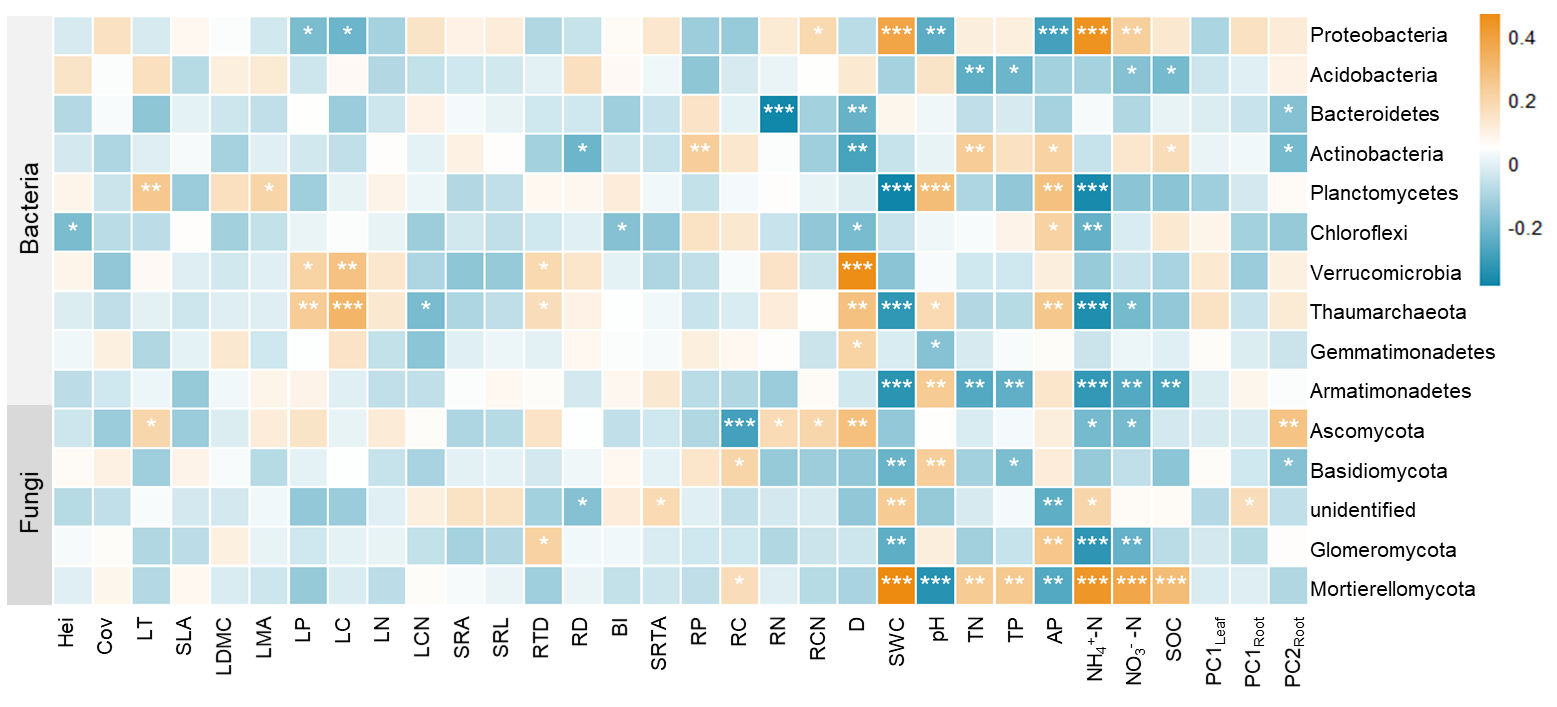


**Figure** **S12** Pearson correlation analysis between the relative abundance of bacteria and fungi with environmental factors in different phyla taxa. The color gradient represents Pearson’s correlation coefficients, and asterisks indicate the significance of Pearson analysis. * : *P* < 0.05; ** : *P* < 0.01; *** : *P* < 0.001. PC1_Leaf_: the conservation gradient of leaf from ‘slow’ to ‘fast’; PC1_Root_: the collaboration strategy of root from ‘outsourcing’ to ‘do-it-yourself’; PC2_Root_: the conservation gradient of root from ‘fast’ to ‘slow’. The detailed description of each soil properties and plant functional traits is presented in Table S1.


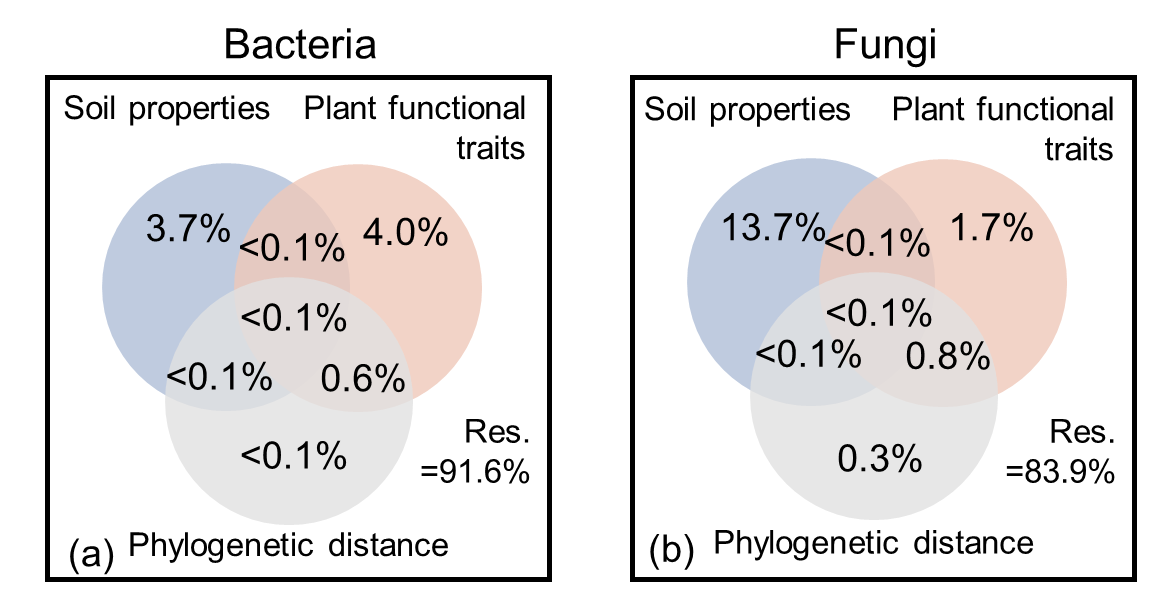


**Figure S13** Variation partitioning for bacterial (a) and fungal (b) community composition based on MRM, explained by selected soil properties, plant functional traits, and phylogenetic distance.
